# Vertebral Metastasis With Syringomyelia Secondary to Breast Adenocarcinoma: A Case Report and Literature Review

**DOI:** 10.1002/ccr3.70580

**Published:** 2025-07-01

**Authors:** Ahmed Reda Abdelhalim, Hossam Tharwat Ali, Bishoy Adel Kamel Zakher, Yara Ashour

**Affiliations:** ^1^ Faculty of Medicine Alexandria University Alexandria Egypt; ^2^ Qena Faculty of Medicine South Valley University Qena Egypt; ^3^ Ain Shams University Cairo Egypt; ^4^ Al‐Quds University Abu Dis Palestine; ^5^ Palestinian Ministry of Health Gaza Palestine

**Keywords:** chemotherapy, intramedullary metastasis, malignancy, radiotherapy, syringomyelia, syrinx

## Abstract

Syringomyelia has several possible causes, commonly including Chiari malformation, spinal cord tumors and injuries, and damage caused by lesions around the spinal cord. A 34‐year‐old female was diagnosed with primary breast cancer and vertebral metastasis and was initiated on an extensive regimen of chemotherapy and radiotherapy. A few months later, she developed numbness and weakness in her lower limbs, and she was concluded to have syringomyelia with surrounding atrophic myelopathy along with vertebral metastasis. Patients developing neurological symptoms following radiotherapy or chemotherapy should be thoroughly evaluated, and further cases should be reported in the literature.

**Abbreviations**
ALT: alanine aminotransferase
AST: aspartate aminotransferase
CBC: complete blood count
CSF: cerebrospinal fluid
CT: computed tomography
ICU: intensive care unit
MRI: magnetic resonance imaging

**Summary**

- Although syringomyelia with non‐neurological malignancies is not common, patients developing neurological symptoms following radiotherapy or chemotherapy, even for non‐neurological malignancies, should be thoroughly evaluated for the presence of syringomyelia.
- Tailored management options should be sought.

## 1 Introduction

Syringomyelia is a neurological disorder in which a fluid‐filled cyst (syrinx) forms within the spinal cord, which can expand enough to damage the spinal cord, causing atrophic myelopathy. Syringomyelia has several possible causes, commonly including Chiari malformation, spinal cord tumors and injuries, and damage caused by lesions around the spinal cord [1, 2]. Additionally, it has been associated with intramedullary metastasis of malignancies of different primary origins [3, 4]. The management of syringomyelia requires a multidisciplinary approach where a thorough evaluation of the patient's risk factors, history, clinical manifestations, and surgical fitness are combined [2, 5].

Some authors suggested that extensive regimens of chemotherapy and radiotherapy can be a cause as well [6, 7]. Yet, there is not enough evidence in the literature to fully conclude radiotherapy and/or chemotherapy as causes of syringomyelia. Similarly, metastatic lytic lesions of the vertebrae have not been strongly linked to the occurrence of syringomyelia [8]. Herein, we report the first case of a young female who had received chemotherapy along with radiotherapy for breast cancer that metastasized to the vertebrae and developed syringomyelia. Furthermore, we have done a review of similar published cases in the literature discussing the common and different points among them [9].

## 2 Case History

A 34‐year‐old female was diagnosed with primary breast cancer (invasive ductal carcinoma Grade 2) and vertebral metastasis. Computed tomography (CT) showed a lytic bony lesion at the eighth thoracic vertebra (T8) and a bone scan showed increased activity at the seventh thoracic vertebra (T7). Chemotherapy and radiotherapy were initiated. The patient received the first doxorubicin (adriamycin) dose followed by 10 radiotherapy sessions. Two days after the last radiotherapy session, she received the second doxorubicin dose. Shortly after, she developed radiation‐induced gastritis and oral candidiasis. The patient then finished the third dose of doxorubicin (304.48 mg in total). Afterward, she finished eight doses of paclitaxel (taxol) along with five doses of trastuzumab (Herceptin) and one dose (Ogivri). After a few months, she developed numbness and weakness in her lower limbs.

## 3 Methods and Investigations

**FIGURE 1:**
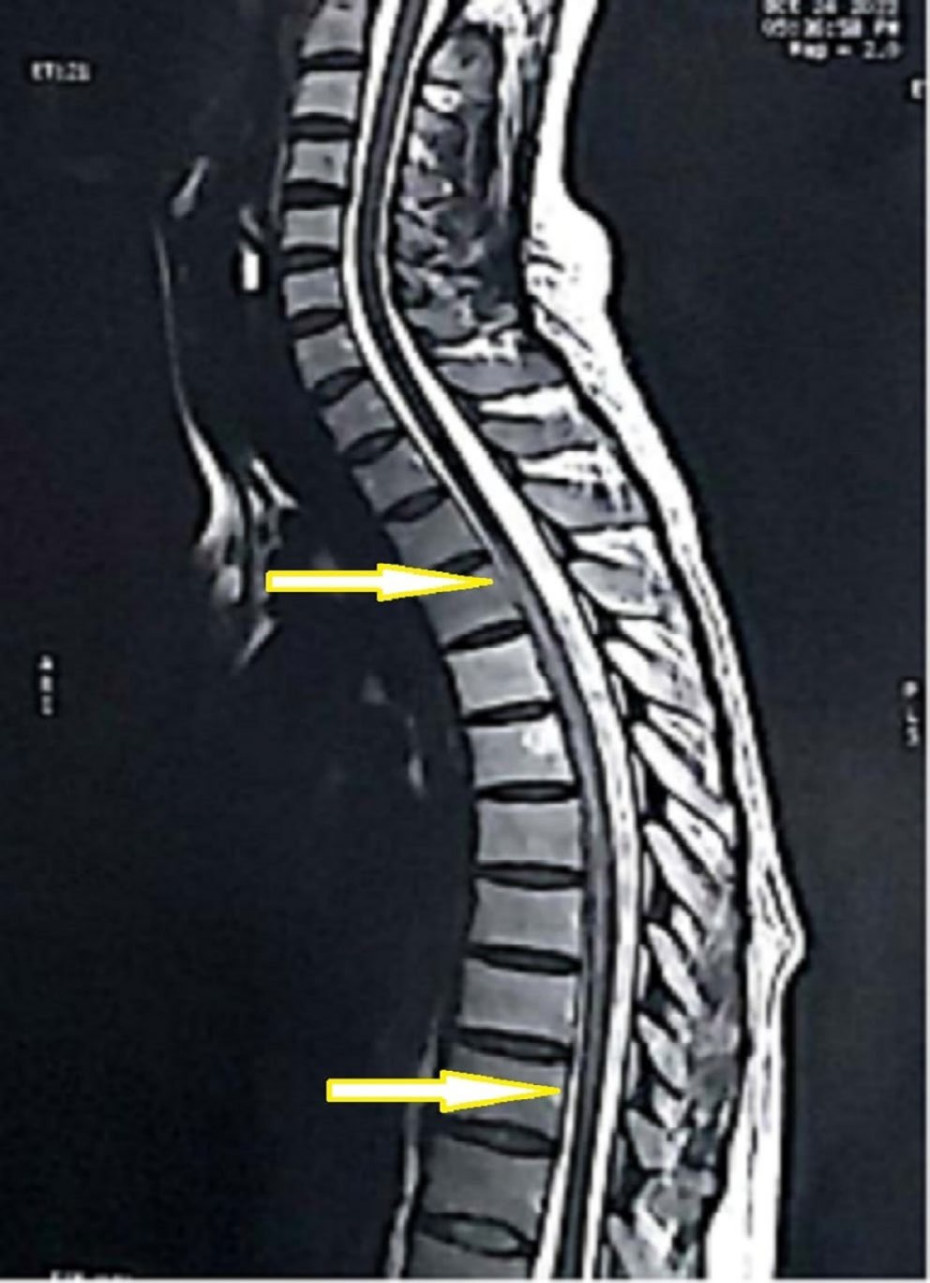
The patient's magnetic resonance imaging, sagittal view of the thoracic vertebrae; the arrows indicate the levels of syringomyelia between the fourth and tenth thoracic vertebrae.

**FIGURE 2:**
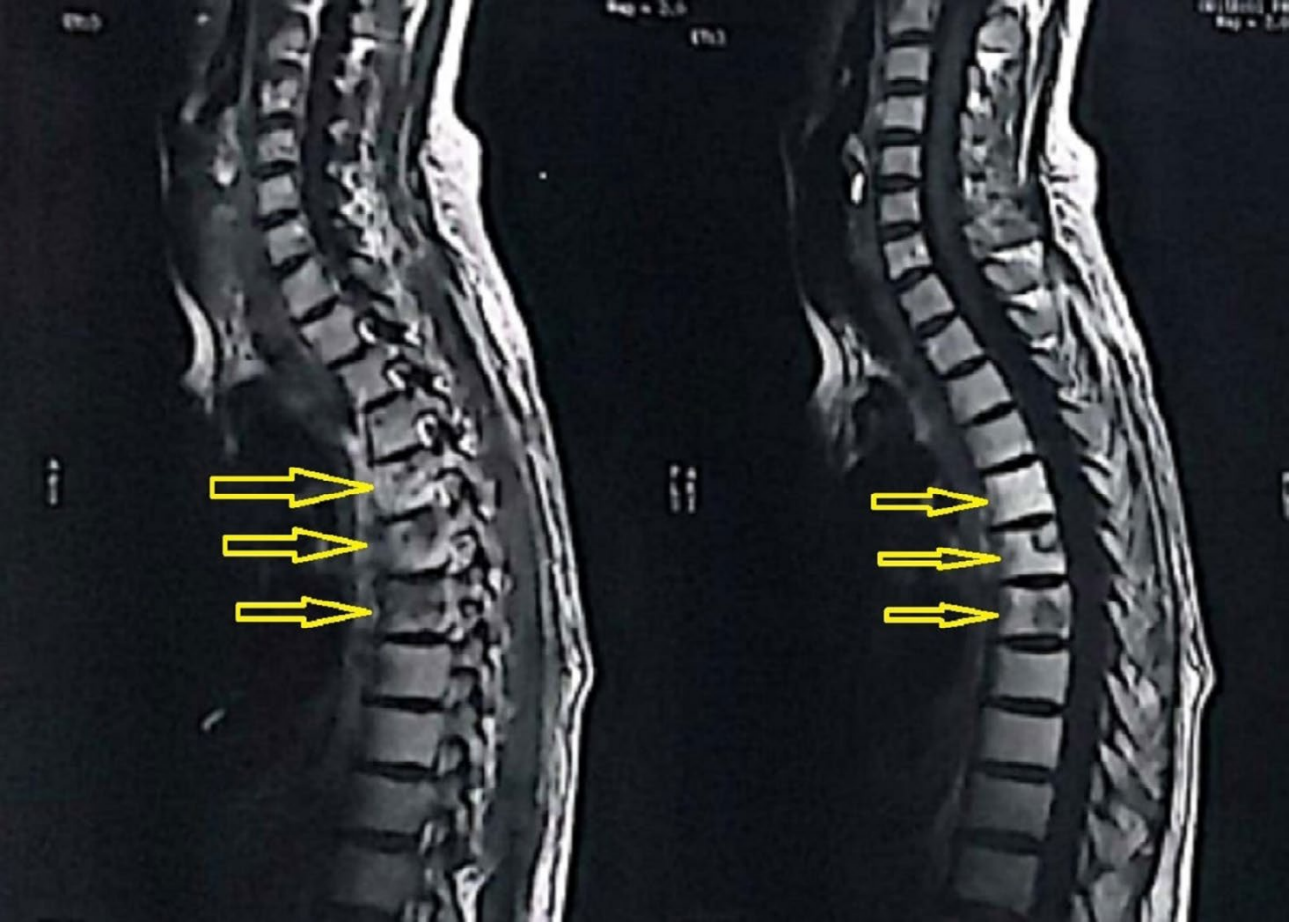
The patient's magnetic resonance imaging, sagittal view of the thoracic vertebrae; the arrows refer to the sixth, seventh, and eight vertebrae with lytic lesions.

**FIGURE 3:**
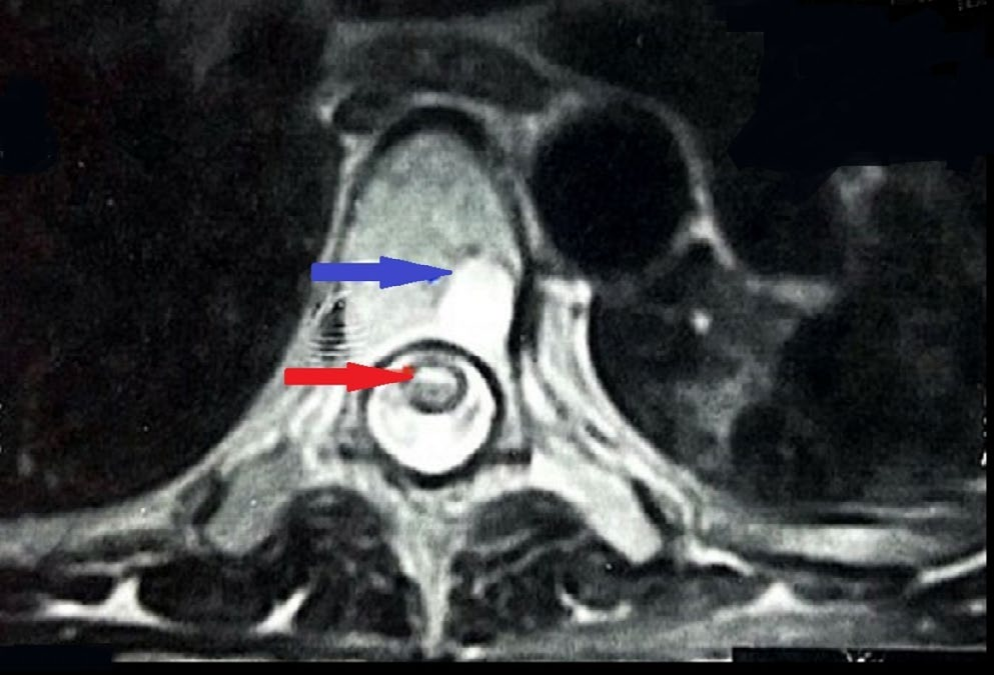
The patient's magnetic resonance imaging, transverse view of the sixth thoracic vertebra, showing both syringomyelia (red arrow) and the body lytic lesion (blue arrow).

She underwent magnetic resonance imaging (MRI) of the cervical, lumber, and dorsal spine. The cervical and lumber MRI were unremarkable; on the other hand, the dorsal MRI showed an appreciable central dilated spinal canal within the spinal cord with reduced remaining spinal cord parenchyma between the fourth and tenth thoracic vertebrae (T4–T10) (Figure 1). It was concluded as syringomyelia with surrounding spinal cord atrophic myelopathy that ended up with paraplegia. Additionally, the MRI showed lytic lesions on the sixth to eighth thoracic vertebrae (T6, T7, and T8) (Figures 2 and 3).

## 4 Results and Outcomes

Shortly afterward, the clinical status deteriorated with abnormal laboratory results. Complete blood count (CBC) showed pancytopenia. Liver function tests (AST and ALT) and renal function tests (urea and creatinine) were elevated. Intensive care unit (ICU) admission was required. She died 48 h after the ICU admission.

## 5 Discussion

Syringomyelia, a longitudinal fluid‐filled cavity inside the spinal cord, can have variable etiologies and underlying mechanisms [2, 5]. It is commonly associated with spinal tumors or structural lesions from the level of foramen magnum [6]. Intramedullary metastasis, although not common, is a possible cause, with lung, breast, colorectal, lymphoma, and melanoma being the common sites for origin [3, 10]. To identify previously reported similar cases of syringomyelia in the context of tumors outside the nervous system, a literature search was carried out on the PubMed database using the following string of keywords: (“Syrinx” OR “syringomyelia” OR “hydromyelia” OR “syringobulbia” OR “syringopontia” OR “syringomesencephaly” OR “syringocephalus”) AND (“chemotherapy” OR “drug” OR “antineoplastic” OR “anticancer” OR “anticancerous” OR “radiotherapy” OR “irradiation” OR “radiation” OR “cancer” OR “neoplasia” OR “neoplasm” OR “Carcinoma” OR “malignant” OR “malignancy” OR “tumor”). A total of 781 results were recruited and screened by two authors independently. Only nine articles describing ten cases were deemed relevant and were described in Table 1.

**TABLE 1:** Previous reported cases of syringomyelia secondary to non‐neurological malignancies, radiation, or chemotherapy.

References	Sex, age (years)	Diagnosis of cancer	Cancer management	Site of syringomyelia	Management of syringomyelia	Follow‐up and special notes
The present case	Female, 34	Primary breast cancer (invasive ductal carcinoma Grade 2) Vertebral metastasis	Chemotherapy involved three doses of doxorubicin (adriamycin) (304.48 mg in total) and eight doses of paclitaxel (taxol) along with five doses of trasuzumab (herceptin) and one dose (Ogivri) Radiotherapy: ten sessions	T4–T10	None #	The patient deteriorated rapidly and died within 48 h of intensive care unit admission
Foster et al. [4]	Male, 66	Stage “C” colonic adenocarcinoma Widespread metastases including thoracic vertebral body and intramedullary metastasis	Anterior resection for the colonic tumor	Upper cervical region	Not explicitly detailed	The patient died due to widespread metastasis
Foster et al. [4]	Female, 55	Breast adenocarcinoma, poorly differentiated Intramedullary metastasis in the spinal cord	Local radiotherapy and oral tamoxifen. After deterioration, a decompressive laminectomy at D6 was performed, revealing a metastatic deposit, which was macroscopically removed. Postoperatively, a second course of radiotherapy was given, followed by rehabilitation	C6–T10	Decompressive laminectomy. Subsequent imaging showed rostral extension of the syrinx, leading to a second surgery where a shunt was inserted into the syrinx, providing some symptomatic improvement	Patient died a few weeks later due to cerebral secondaries
Ostler et al. [6]	Male, 37	Poorly differentiated squamous cell carcinoma (oropharyngeal carcinoma)	Primary chemotherapy involved two cycles of carboplatin and 5‐fluorouracil + radical external beam radiotherapy + surgical intervention followed by Iridium‐192 wire brachytherapy	C2–C5	Syrinx was deemed not suitable for surgical intervention	Although the management of oropharyngeal tumor was completed, the neurological deficit attributed to syringomyelia significantly impacted the patient's quality of life, leading to medical retirement. The patient was a heavy alcoholic
Shindo et al. [7]	Male, 70	Right upper lung adenocarcinoma	Radiation therapy (48 Gy) on neck and mediastinum	T2–T5	Not explicitly detailed	The patient developed neurological symptoms eight years after the radiation therapy
Poggi et al. [8]	Male, 68	Undifferentiated small‐cell lung cancer Metastasis to T1 vertebral body a week later	Chemotherapy (not detailed)	T3–T6	None #	The patient's neurological manifestations improved. Palliative radiotherapy to the spinal cord was considered but ultimately deferred since the syringohydromyelia did not appear to have a malignant etiology and there was no evidence of cord compression due to the cancer
Phuphanich et al. [10]	Male, 42	Well‐differentiated small‐cell lung adenocarcinoma Metastasis to cerebellopontine angle, brain, and then intramedullary	Specific treatment for lung adenocarcinoma is not detailed Metastasis: whole‐brain and spinal cord radiation therapy course of 3000 cGy	C4–T10	None #	The patient passed away two months later
Ashawesh et al. [11]	Male, 68	Small‐cell cancer of the left lung Intramedullary spinal cord metastasis and syringomyelia 4 months later	Palliative chemotherapy and radiotherapy for the primary lung cancer Urgent radiotherapy applied to spinal cord metastasis	C2–T1	None #	Spinal cord radiotherapy led to an improvement in the neurological manifestations and the quality of life
Zhou et al. [12]	Male, 79	Primary colorectal diffuse large B‐cell lymphoma Cerebellar metastasis one year later Metastatic spinal intramedullary malignant lymphoma 6 months later	Ileocecal tumor resection and postoperative chemotherapy with cyclophosphamide, doxorubicin, vincristine, and prednisone (CHOP) plan for 13 cycles For cerebellar metastasis, another 6 cycles of systemic chemotherapy and underwent several courses of local radiotherapy	T2–L3	Spinal cord radiotherapy and systemic chemotherapy with intrathecal injections of methotrexate	Patient died of multiple organ failure 3 months later
van Uum et al. [13]	Female, 20	Malignant prolactinoma Metastasis to the leptomeninges extending from the brainstem to L5 with thoracic syringomyelia 30 years later	Occipito‐cervical decompression High‐dose cabergoline therapy	T7–T8	Thoracic laminectomy at T7 with the insertion of a syringo‐subarachnoidal cerebrospinal fluid (CSF) shunt to alleviate the blockage of CSF flow	Although the tumor load decreased despite these interventions, the patient's walking difficulties and sensory disturbances did not improve post‐surgery, and she remained wheelchair dependent
Phuphanich et al. [14]	Female, 44	Cervical cord astrocytoma	4500 cGy of local radiation therapy to the cervical cord (C1–C5) followed by surgery to remove the tumor. Post‐surgery, the patient underwent chemotherapy and interferon treatment with the development of what was thought to be a recurrent tumor. This was later confirmed not to be a recurrent tumor but rather radiation necrosis with evidence of multiple sclerosis	T4–T10	Chemotherapy was initiated as the syrinx was thought to be a cyst of tumor origin; however, it was diagnosed as radiation necrosis with cyst formation on autopsy	The patient died due to respiratory failure

Of the tabled cases, five were associated with intramedullary metastasis due to lung cancer (two cases) [10, 11], colon cancer (one case) [4], breast cancer (one case) [4], and lymphoma (one case) [12]. In such cases, syrinx involved the level of the cervical and/or thoracic spine. Not only intramedullary metastasis but also vertebral and leptomeningeal metastases that syrinx were reported within previous cases [8, 13]. The present case encompassed a syrinx between the level of the fourth and tenth thoracic vertebrae along with lytic lesions, mostly metastatic lesions from the breast cancer, at the sixth, seventh, and eighth thoracic vertebra. Similarly, a previous case of lung cancer developed vertebral metastasis at the first thoracic vertebra along with a syrinx between the third and sixth thoracic vertebrae [8]. Interestingly, van Uum et al. [13] reported a case with prolactinoma that metastasized to the leptomeninges extending from the brainstem to the fifth lumbar vertebra and associated with a syrinx at the level of the seventh and eighth thoracic vertebrae. While the present case is considered an unusual site for breast cancer metastasis, it is advisable to consider unusual sites of metastasis, especially in complicated cases with an unusual course of disease, and the report of such cases should always be encouraged [9, 15, 16] as well as advanced imaging and screening programs for early detection and better survival of cancer cases, especially in countries where certain cancers are prevalent [17, 18].

Despite the clinical manifestations of the syrinxes, in most cases, seven cases including the present one, there was not a specific treatment or intervention to improve the syrinx or alleviate the associated symptoms. Two cases showed symptomatic improvement following decompressive laminectomy and insertion of syringo‐subarachnoidal cerebrospinal fluid (CSF) shunts [4, 13]. Chemotherapy was tried in two cases [12, 14], while spinal radiotherapy was done in only one case [12].

Although local radiotherapy has been suggested as a treatment option for syringomyelia, some authors argue that syringomyelia can occur as a radiotherapy‐related complication [6]. It is noteworthy that radiotherapy was initiated for malignancies in seven cases including ours [4, 6, 7, 10, 11, 14]. In the case reported by Phuphanich et al. [14], a postmortem examination revealed the syrinx as radiation necrosis rather than a cyst of neoplastic origin. In two cases who had malignancies of primary non‐neurological origins, lung and oropharyngeal carcinomas, with no evidence of either intramedullary or vertebral metastases, the patients developed syringomyelia and neurological symptoms [6, 7]. In one case, chemotherapy and radiotherapy were initiated [6], while only radiotherapy was done for the other [7] as treatment for the primary malignancy.

Notably, only in two case [4, 13], surgical options were attempted in terms of decompressive laminectomy with shunt insertion which provided symptomatic relief. In the case reported by Phuphanich et al. [14], chemotherapy was initiated as the physicians believed it was a cyst of neoplastic origin which later were correctly diagnosed as radiation necrosis in autopsy. Similarly, Zhou et al. [12] reported using spinal cord radiotherapy, intrathecal chemotherapy along with the systemic chemotherapy, most probably as they believed the spinal lesion was a cancerous lesion. The rest of cases did not attempt specific management for syringomyelia. It is essential to consider that the reported cases exhibit complicated cases with rare complication of the syringomyelia which can be challenging to provide comprehensive and timely management.

While the exact mechanism for syringomyelia occurrence is not clear, with some suggested hypotheses for common congenital cases [1, 19], the underlying pathogenesis related to chemotherapy and radiotherapy needs to be extensively studied. For instance, radiation can cause wide changes in the spinal cord, including reactive gliosis, parenchymal necrosis, and disruption of the blood–spinal cord barrier, which all can predispose to CSF circulation disruption and formation of the syrinx [19, 20]. The addition of chemotherapy can increase the possibility of parenchymal necrosis due to multiple metabolic apoptotic pathways [21, 22]. Animal studies also showed that chemotherapy can induce vascular permeability change in the blood–spinal cord barrier [22, 23]. It is noted that in our case, although there was evidence for vertebral metastasis, the role of radiotherapy and chemotherapy in the pathogenesis of syringomyelia cannot be excluded and yet cannot be established definitively. More cases and studies are encouraged to report a definitive link between syringomyelia and the management of non‐neurological cancers.

In conclusion, syringomyelia can have variable etiologies and underlying mechanisms. The present case represents a young female who developed syringomyelia and vertebral metastasis secondary to breast adenocarcinoma. We believe there is no sufficient evidence of syringomyelia with non‐neurological malignancies to fully understand the pathogenesis of syringomyelia in such cases. Patients developing neurological symptoms following radiotherapy or chemotherapy should be thoroughly evaluated and further cases should be reported in the literature.

## Author Contributions

**Ahmed Reda Abdelhalim:** conceptualization, investigation, methodology, writing – original draft. **Hossam Tharwat Ali:** data curation, methodology, writing – review and editing. **Bishoy Adel Kamel Zakher:** data curation, writing – original draft. **Yara Ashour:** data curation, writing – original draft.

## Ethics Statement

The study was done in accordance with the ethical standards of the 1964 Helsinki Declaration. Ethics approval was waived by the local committee because no personal data or image was used.

## Consent

Informed consent was taken from the patient's husband for this study. Written informed consent was obtained from the patient's husband to publish this case report.

## Conflicts of Interest

The authors declare no conflicts of interest.

## Data Availability

The datasets used and/or analyzed during the current study are available from the corresponding author on reasonable request.
